# Detection of Reduced Diameter of the Cochlear Nerve in Long-term Deaf Patients Quantified With Semiautomatic Measurement of Nerve Cross-sectional Area Using 3T MRI Data

**DOI:** 10.1097/ONO.0000000000000047

**Published:** 2024-01-25

**Authors:** Katrin Reimann, Uwe Klose, Ulrike Ehrenpfordt, Kruthika Thangavelu, Maximilian Schulze

**Affiliations:** 1 Department of Otolaryngology, Head and Neck Surgery, University of Tübingen, Tübingen, Germany; 2 Department of Neuroradiology, University of Tübingen, Tübingen, Germany; 3 Department of Otorhinolaryngology, Head and Neck Surgery, University Hospital Marburg, Marburg, Germany

**Keywords:** 3T MRI, Nerve cross-sectional area, Semiautomatic measurement, SHNL, presbyvertigo, pTX T2 SPACE, vestibular nerve

## Abstract

**Hypothesis:**

High-resolution parallel transmit T2 sampling perfection with application optimized contrast using different flip angle evolution sequence with improved edge discrimination and semiautomatic determination of nerve cross-sectional area (CSA) can be used to evaluate nerve degeneration in the inner auditory canal (IAC) in long-term deaf patients.

**Background:**

In patients with hearing loss, temporal bone MRI is routinely acquired to evaluate the morphology of the nerves within the IAC. Earlier studies have shown that the diameter of the cochlear nerve can be used as prognostic marker for the auditory performance after cochlear implantation in postlingually deaf patients.

**Methods:**

Eighty-two consecutive MRI scans were analyzed using a semiautomatic tool to measure CSA of cranial nerves in the IAC. Results were correlated with patient history and audiology testing as well as with age and gender.

**Results:**

There was a significant reduced CSA of the cochlear nerve in ears with moderate-to-profound hearing loss and deafness compared with ears with normal hearing, but no significant difference in ears with mild-to-moderate hearing loss compared with normal hearing. In detail, normal hearing ears had a CSA of 1.23 ± 0.11 mm^2^, whereas ears with pantonal hearing loss of more than 40 dB had 1.02 ± 0.05 mm^2^ (*P* = 0.026). Maximal CSA of the facial nerve was not different among all groups (average, 1.04 mm^2^ ± 0.03; linear regression, *P* = 0.001) and stable with age. However, vestibular nerve CSA decreased significantly with age (average, 1.78 ± 0.05 mm^2^; linear regression, *P* = 0.128).

**Conclusions:**

In long-term deaf patients, smaller the diameter of cochlear nerve is the more severe the hearing loss is. The new semiautomatic tool can primarily be used to assess nerve diameter and possibly determine ears with nerve degeneration.

Patients with profound sensorineural hearing loss (SNHL) or deafness are routinely treated with cochlear implantation (CI) to restore hearing (1). Besides audiological testing, routine preoperative evaluation includes MRI scan of the temporal bone and neurocranium. MRI enables the evaluation of the structures of the inner ear, as well as the morphology of the nerves in the inner auditory canal (IAC). Earlier studies have shown that the diameter of the cochlear nerve (CN) can be used as prognostic marker for the auditory performance after CI in postlingually deaf patients (2). In children with hereditary severe SNHL of unknown origin, as well as in children with connexin 26 mutation, MRI examination could demonstrate a small hypoplasia of the CN compared with healthy children with normal hearing (3). However, Sildiroglu et al. (4) found no difference in CN diameter in older patients with SNHL, compared with young patients with normal hearing. An anatomical study by Nadol and Xu (5) showed that the maximum diameter of the CN was smaller in deaf patients than in patients with normal hearing. Using 1.5T MRI, Herman and Angeli (6) could confirm this finding measuring significant smaller CN cross-sectional area (CSA) in postlingually deaf patients. Measurements of nerve diameter in earlier studies showed that in most ears (88%), CN is normal whenever its diameter is larger or at least equal to that of the facial nerve (FN) (7). In all previous studies, measurement of nerve diameter was done by hand. Additionally, there were differences in image acquisition (1.5T MRI, 3T MRI, MRI sequence) and image resolution.

The purpose of this study was to use high-resolution parallel transmit (pTX) T2 sampling perfection with application optimized contrast using different flip angle evolution (SPACE) sequence (8,9) with improved edge discrimination and semiautomatic determination of nerve CSA, using an algorithm that excludes partial volume effect by only including voxels of a defined signal strength. Therefore, we analyzed patients with 3T MRI and correlated the CSA of the nerves in the IAC with the audiological testing and patient history (length and characteristics of hearing loss), as well as with gender and age.

## MATERIALS AND METHODS

### Data Acquisition

The study was approved by the local ethics committee. Written informed consent was obtained from each patient before enrollment into the study. From October 2013 to October 2016, patients who had a routine high-resolution temporal bone MRI due to hearing loss, acquired at 3 T with the recently published pTX T2 SPACE sequence (9), were enrolled in the study. Eighty-two patients were included in the study depending on the patient history for hearing loss (more than 10 years) and audiogram (pantonal SNHL, left corner hearing loss). Patients with a history of FN palsy or loss of vestibular function, and patients with conductive or mixed hearing loss were excluded. Patient groups were determined according to the audiogram at the time of the MRI. Normal hearing contralateral ears in patients with unilateral hearing loss served as normal hearing control group.

### Patient Groups

For evaluation of FN and vestibular nerve (VN), only patients without a history of facial palsy or loss of vestibular function were included. About 155 temporal bones were included to evaluate the average CSA of the FN and 156 were included to evaluate the VN.

**FIG. 1.**
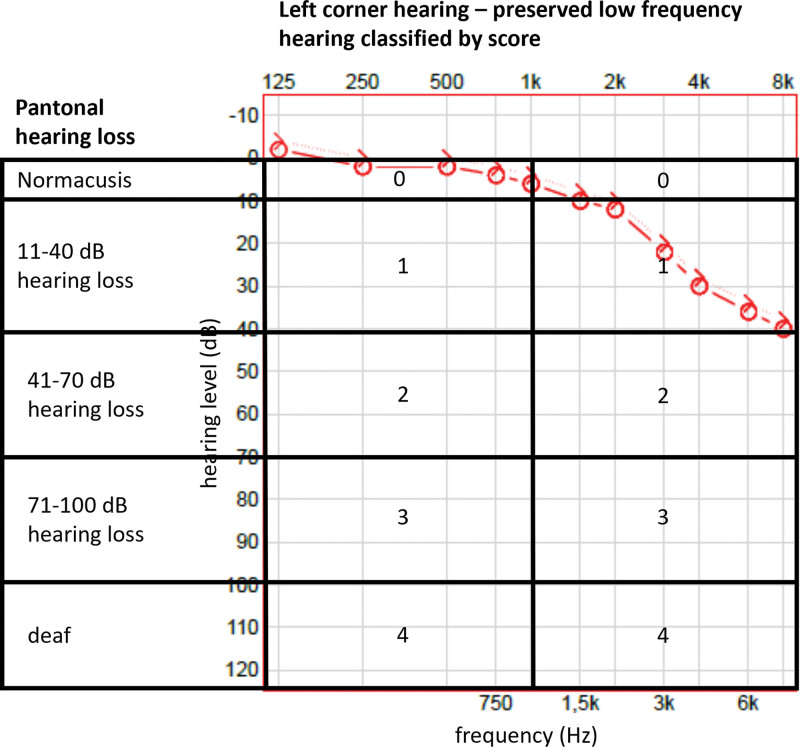
Schematic illustration of hearing evaluation for patients groups with pantonal hearing loss and left corner hearing.

1. Pantonal hearing loss: Patients were grouped according to results of audiometric measurements in normal hearing ears (0–10 dB hearing loss), ears with mild-to-moderate hearing loss (11–40 dB), moderate-to-profound hearing loss (41–70 dB), profound hearing loss (71–100 dB), and deafness (101–120 dB), respectively (Fig. 1). Seventy-nine temporal bones were evaluated in this group.
2. “Left corner” hearing: This describes ears with diagonally shaped audiograms, with better hearing in low frequencies and severe hearing loss in high frequencies. The purpose is to evaluate whether better hearing in low frequencies can prevent CN degeneration, compared with pantonal hearing loss. Ears were grouped according to results of audiometric measurements. Audiograms were divided into 5 groups, and each group received a score depending on the severity of the hearing loss (normal = 0; 11–40 dB = 1; 41–70 dB = 2; 71–100 dB = 3; deaf = 4). To accommodate for left corner hearing, the audiogram was split at 1 kHz. To determine the hearing impairment score, the left side score and the right side score were added, thus the higher the value/score, the more severe the hearing loss (Fig. 1).

### MRI Acquisition Using pTX T2 SPACE Sequence

For MRI protocol and examinations, all MRI images were acquired using a 3T whole-body MRI system (MAGNETOM Skyra 3 T, Siemens Healthcare, Erlangen, Germany) using 2 independent coils for transmitting and a 20-channel head/neck coil for receiving. A clinical routine temporal bone MRI protocol containing a three-dimensional (3D) T2 pTX SPACE sequence was obtained in all patients. pTX T2 SPACE acquisition enclosed the skull base in the anteroposterior direction from the apex of the petromastoid bone to the occipitomastoid suture.

Imaging parameters for pTX SPACE were as follows: 64 partitions in axial orientation, 1000 ms repetition time, 136 ms echo time; 124 × 320 acquisition matrix and 248 × 640 reconstructed matrix, 130 mm field of view, 256 Hz/Px bandwidth, 0.2 mm × 0.2 mm × 0.4 mm voxel size after interpolation; 4 min 22 s acquisition time (9). MRI images were transferred to a dedicated Leonardo workstation (Siemens Healthcare, Erlangen, Germany), and oblique sagittal reconstructions through the IAC were acquired, perpendicular to the IAC, where the cranial nerves in the IAC, FN, CN, superior, and inferior branch of the VN could be identified.

### Semiautomatic Determination of Nerve Cross-sectional Area (CSA) of Cochlear and Facial Nerves

Evaluation was done with a dedicated MATLAB program, which was developed for this task (The Mathworks, Inc, Natick, MA). Parasagittal reconstructions through the IAC were acquired, perpendicular to the cranial nerves in the IAC. To determine the CSA of the FN and the CN, a representative section was chosen, in which all 4 cranial nerves (FN, CN, superior, and inferior branch of the VN) were seen. Within this section, 4 regions of interest (ROIs) were interactively selected.

To determinate the signal of the nerve without influence of the surrounding cerebrospinal fluid (CSF), a ROI 1 was placed within the CN; while taking care, that the ROI 1 was located well within the nerve, distant to the edge of the nerve, to avoid including the area of partial volume at the transition zone from nerve to CSF.

To determine the signal within the CSF, a ROI 2 was placed within the IAC in the brightest area of the CSF.

One ROI 3 was placed within the CN, and another ROI 4 was placed within the FN, along the transition zone from nerve to CSF, that is, along the margin of which the hypointense signal of the nerve transits into hyperintense signal of the CSF. Thus, deliberately the zone, in which partial volume effects are present, was selected within the ROI. In those few cases, where a part of the nerve was located directly beside the osseous wall of the IAC, with no CSF lamella between bone and nerve, the margin of the nerve was marked, according to the nerve course, known from the whole dataset.

As characteristic signal within the nerve and the CSF, the median of the signal distributions within ROIs 1 and 2 were calculated, *S*_nerve_ and *S*_CSF_. The mean of both values denotes the signal value *S*_50_, at which the portion of the CSF regarding the signal of the particular voxel exceeds 50% (Equation 1)

*S*_50_ = (*S*_CSF_ + *S*_nerve_)/2 (1).

In regions 3 and 4, all pixels were deleted, which held a signal intensity larger than *S*_50._

**FIG. 2.**
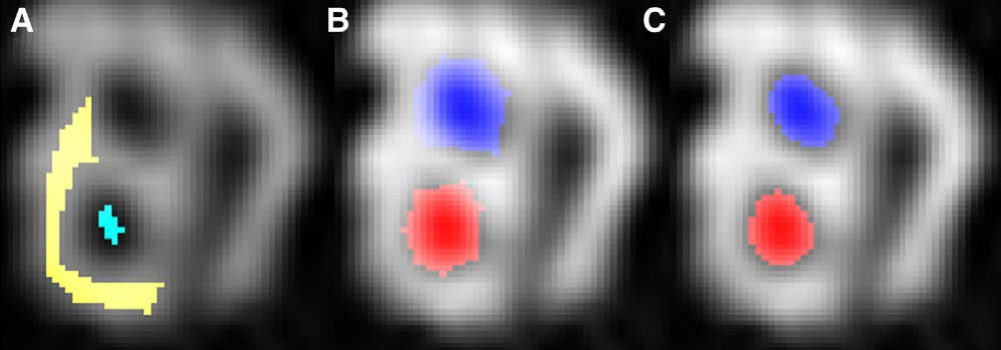
Example of semiautomatic quantification of cochlear and facial nerve on parasagittal reconstruction of IAC. *A*, ROI 1, in cyan, placed within the cochlear nerve and ROI 2, in yellow, placed within the IAC in the brightest area of the CSF. *B*, ROI 3 and ROI 4 placed within the cochlear and facial nerve along the transition zone from nerve to CSF. *C*, Areas of both cross-sectional areas of the nerves that were calculated. CSF indicates cerebrospinal fluid; IAC, inner auditory canal; ROI, region of interest.

From the number of remaining pixels, considering the spatial extent of a pixel (0.102 × 0.102 mm^2^), the area of both CSAs of the nerves were calculated (Fig. 2). Finally, we calculated the ratio of the CSA of CN and VN, to accommodate for the varying sizes of the internal auditory canal and its nerves.

### Statistical Analysis

Two-sample *t* test was used to compare CSA of CN, FN, and VN between genders. The CSA of CN across various degrees of hearing impairment was compared with normal hearing, and 2-sample *t* test, assuming equal variances, was done; *P* value was calculated for the same in patients with pantonal hearing loss. Similarly, 2-sample *t* test was used to check significant differences between CN CSA across various hearing impairment scores, when compared with normal hearing patients.

Linear regression analysis was done, comparing age and CSA of the VN und FN. A *P* value <0.05 was considered statistically significant.

Statistical computations were performed using JMP 11.2.0, SAS Institute Inc., Böblingen, Germany.

## RESULTS

### Patient Groups

1. Pantonal hearing loss: Patient group characteristics are shown in Table 1. The mean age of all patients was 52.81 ± 1.93 years. The mean duration of hearing loss, according to patient history, was 22.29 ± 1.78 years. There was no significant difference in the duration of hearing loss among groups 3–5. Distribution of gender was also balanced within groups. Seventy-nine temporal bones were evaluated in this group.
2. “Left corner” hearing: Patient group characteristics are shown in Table 2.The mean age of all patients was 41.85 ± 2.06 years. The mean duration of hearing loss according to patient history was 24.62 ± 1.69 years. There was no significant difference in the duration of hearing loss among groups 3–7. Distribution of gender was also balanced within groups. Seventy-one temporal bones were evaluated in this group.

**TABLE 1.** Patient group characteristics with pantonal hearing loss

Patient groups		Patient history	
1	Control n = 10	Age (years); mean ± SEM Gender (female/male)	33.4 ± 4.41 5f/5m
2	11–40 dB hearing loss n = 13	Age (years); mean ± SEM Gender (female/male)	56.85 ± 4.48 6f/7m
3	41–70 dB hearing loss n = 21	Age (years); mean ± SEM Gender (female/male) Hearing loss (years); mean ± SEM	60.57 ± 2.61 10f/11m 20.41 ± 3.15
4	71–100 dB hearing loss n = 22	Age (years); mean ± SEM Gender (female/male) Hearing loss (years); mean ± SEM	55.27 ± 3.21 13f/9m 22.4 ± 2.6
5	Deafness n = 13	Age (years); mean ± SEM Gender (female/male) Hearing loss (years); mean ± SEM	47 ± 5.52 5f/8m 26.31 ± 3.78

n represents evaluated temporal bones.

f indicates female; m, male; SEM, standard error of the mean.

**TABLE 2.** Patient group characteristics with left corner hearing loss

Patient groups		Patient history	
1	Control n = 10	Age (years); mean ± SEM Gender (female/male)	33.4 ± 4.41 5f/5m
2	1 hearing score n = 6	Age (years); mean ± SEM Gender (female/male) Hearing loss (years); mean ± SEM	47.83 ± 5.83 2f/4m 17.5 ± 4.29
3	3 hearing score n = 13	Age (years); mean ± SEM Gender (female/male) Hearing loss (years); mean ± SEM	67.62 ± 3.05 7f/8m 22.54 ± 3.63
4	5 hearing score n = 15	Age (years); mean ± SEM Gender (female/male) Hearing loss (years); mean ± SEM	60.2 ± 3.45 4f/6m 22.53 ± 2.88
5	6 hearing score n = 10	Age (years); mean ± SEM Gender (female/male) Hearing loss (years); mean ± SEM	48.4 ± 5.43 4f/6m 29.7 ± 4.8
6	7 hearing score n = 17	Age (years); mean ± SEM Gender (female/male) Hearing loss (years); mean ± SEM	57.76 ± 4.34 8f/9m 27.59 ± 3.47

f indicates female; m, male; SEM, standard error of the mean.

### Average Cross-sectional Area of Facial, Vestibular, and Cochlear Nerves in Healthy Patients

**FIG. 3.**
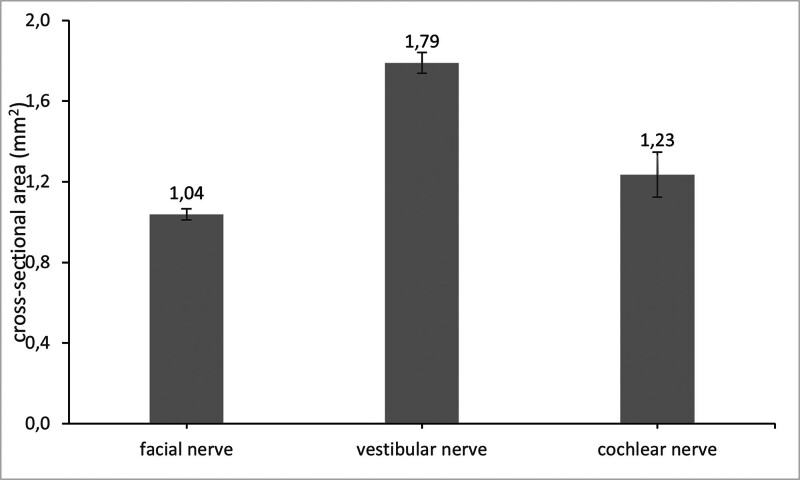
Average of cross-sectional area of facial nerve (n = 155), vestibular nerve (n = 156), and cochlear nerve (n = 10) in mm²; error bars indicate SEM (standard error of the mean).

Using the semiautomatic quantification algorithm, we evaluated 155 temporal bones from patients without a history of FN palsy, to determine the normal CSA of the FN in the internal auditory canal. The average CSA for the FN was 1.04 ± 0.03 mm^2^. For the VN, we evaluated 156 temporal bones. The average CSA of the VN was 1.78 ± 0.05 mm^2^. For the CN, we included 10 temporal bones with normal hearing. The average CSA of the CN was 1.12 ± 0.11 mm^2^ (Fig. 3).

### Influence of Age and Gender on Cross-sectional Area of Facial, Vestibular, and Cochlear Nerves

**FIG. 4.**
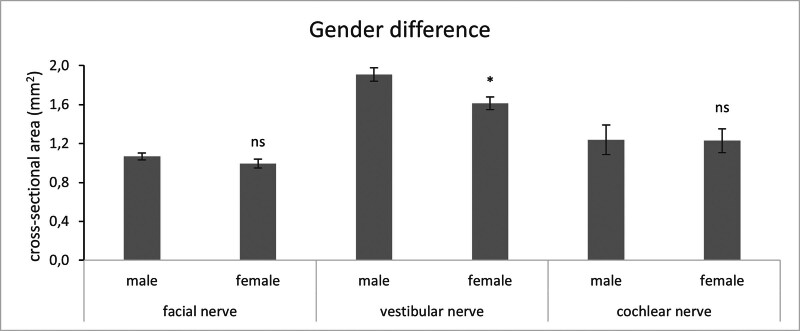
Average of cross-sectional area of facial nerve in males (n = 92) and females (n = 63), vestibular nerve in males (n = 92) and females (n = 63), and cochlear nerve in males (n = 5) and females (n = 5) in mm²; error bars indicate SEM (standard error of the mean).

The average age at the time of MRI of all included female patients (n = 32) was 55 ± 2.44 years, and 55 ± 2.82 years for male patients (n = 48) and therefore similar. There was no difference between the gender in FN, and CN CSA. However, we found that there was a significant smaller CSA of the VN in women (male, 1.91 ± 0.07 mm^2^ and female, 1.62 ± 0.07 mm^2^; *P* = 0.001; Fig. 4).

**FIG. 5.**
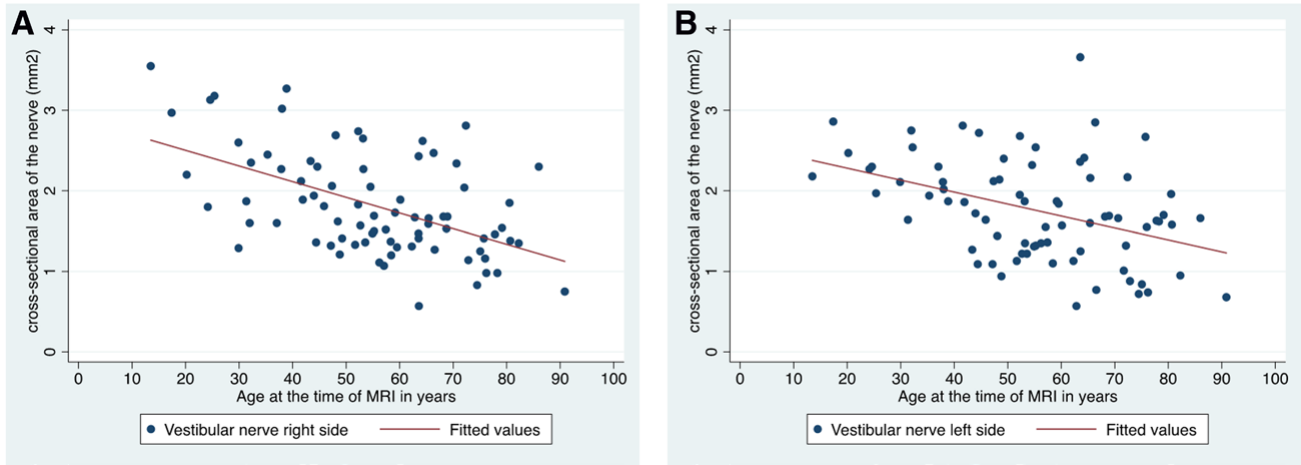
Scatter plot and regression of the cross-sectional area of the vestibular nerve in mm²; line showing the effect of age. *A*, Right side (*P* value <0.001). *B*, Left side (*P* value = 0.001).

**FIG. 6.**
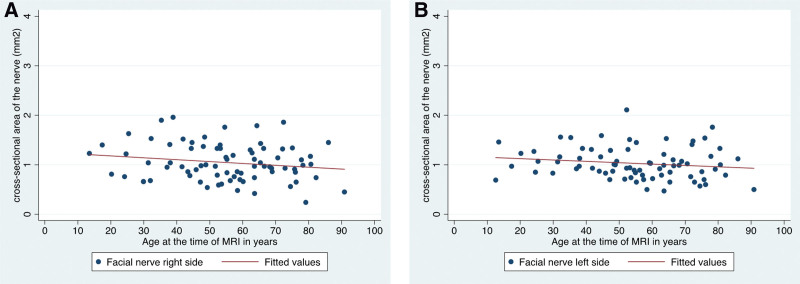
Scatter plot and regression of the cross-sectional area of the facial nerve in mm²; line showing the effect of age. *A*, Right side (*P* value = 0.115). *B*, Left side (*P* value = 0.178).

Multiple linear regression was used to test if age was significantly associated with the CSA of the VN and FN. The fitted regression model was: age = 74.5 − 18.8 (VN) + 13.4 (FN). The overall regression was statistically significant (*R*^2^ = 0.28, *F*_(2,77)_ = 14.7, *P* = 0.000). Interestingly, we found a significant decrease in VN CSA in correlation with the patient’s age (linear regression: *β* = −18.8, 95% confidence interval, −26.3 to −11.4, *P* = 0.000). This effect was observed on both ears of the patients (Fig. 5). There was no age dependence of FN CSA (linear regression: *β* = 13.4, 95% confidence interval, −0.5 to 27.4, *P* = 0.059). This was also true for either side (left and right ears, Fig. 6).

### Reduced Diameter of Cochlear Nerve in Long-time Deaf Patients With Pantonal Hearing Loss

Using the semiautomatic quantification algorithm, we could show that there was a significant reduction in CN CSA between patients with normal hearing and patients with hearing loss of more than 40 dB for more than 10 years. In detail, normal hearing patients had a CSA of 1.23 ± 0.11 mm^2^, whereas patients with pantonal hearing loss of more than 40 dB had 1.02 ± 0.05 mm^2^. The more severe the hearing loss, the smaller was the CN CSA in relation to the FN.

**FIG. 7.**
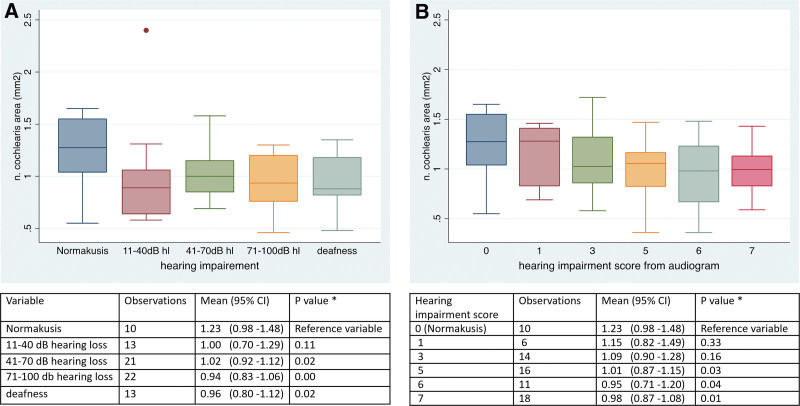
Boxplots of cross-sectional area of cochlear nerve in mm² in normal hearing patients (n = 10) and patients with long-term SNHL. *A*, With pantonal hearing loss: 11–40 dB (n = 13), 41–70 dB hearing loss (n = 21), 71–100 dB (n = 22) hearing loss and deafness (10). *B*, With left corner hearing. Hearing impairment score 0 equals normal hearing (n = 10), 1 (n = 6), score 3 (n = 13), score 5 (n = 15), score 6 (n = 10), score 7 (n = 17). SNHL indicates sensorineural hearing loss.

However, there was no significant difference in patients with moderate hearing loss (between 11 and 40 dB) (Fig. 7A). Therefore, we could show that in long-term deaf patients, smaller the CSA of the CN is the more severe the hearing loss is.

### Reduced Diameter of the Cochlear Nerve in Long-time Deaf Patients With Left Corner Hearing Loss

To evaluate the CSA in patients with left corner hearing loss, the hearing impairment score was calculated, as described above. Six groups with at least 5 temporal bones were formed, which had to be evaluated. We found that there was a significant reduction in CN CSA with hearing impairment score of 5, 6, or 7. There was no significant difference between the CSA of normal hearing patients and patients with hearing impairment scores of 1 and 3, respectively. In detail, CSA decreased from 1.23 ± 0.11 for normal hearing patients to 0.98 ± 0.06, for score 5 patients to 0.96 ± 0.04, and for score 6 patients (Fig. 7B). Parallel to the results in patients with long-lasting pantonal hearing loss, CN CSA is decreasing significantly the more severe the hearing loss. However, there is no significant difference in patients with moderate hearing loss.

## DISCUSSION

We could show that, depending on the severity of hearing loss, the CSA of the CN is decreasing from normal hearing patients to deaf patients. This is in line with a cadaver study, which showed significantly smaller CN diameters in the deaf population, compared with the normal hearing group (5). However, due to dehydration of the CN in the fixed sample, absolute values of nerve diameter have to be carefully appreciated. Earlier studies, measuring nerve diameter on MRI images, are restricted by their measurement method, as measurements were done solely manually (4,7,11). Therefore, one goal of our study was to test a semiautomatic algorithm to measure nerve CSA on 3T pTX T2 SPACE images, using a signal value *S*_50_, at which the portion of the CSF, regarding the signal of the particular voxel, exceeds 50% to exclude voxels with a strong CSF partial volume from the evaluation. For comparison, Nakamichi et al. (11) measured FN and CN diameters in normal hearing patients manually using the so-called full-width at half-maximum method, with the nerve boundary defined at the level of 50% intensity between minimum (nerve) and maximum (CSF). Image acquisition was done using 3T MRI 3D CISS sequence. They measured the CN diameter and calculated a CN CSA of 1.07 ± 0.3 mm^2^. For the FN, they calculated a FN CSA of 0.83 ± 0.27 mm^2^. This is both somewhat smaller compared with our results using the semiautomatic quantification algorithm together with 3T MRT pTX T2 SPACE imaging. The average FN CSA in our study was 1.04 ± 0.03 mm^2^ and 1.23 ± 0.11 mm^2^ for the CN (Table 3). For the VN, Polat et al. (13) report an average CSA of 0.87 ± 0.37 mm^2^ using 1.5T MRI 3D FIESTA sequence. To improve images, a resizing procedure was implemented using Lanczos-2 kernel (4), and by calculating a threshold using Otsu’s method (5). A radiologist then denoted the structures of the IAC, and the pixels of the object in the image were counted to calculate the area of the selected structures of the IAC. In contrast to that, we measured a VN CSA of 1.78 ± 0.05 mm^2^. However, in this study, FN and CN CSA are also significantly smaller compared with our results. In total, we found 7 studies that aimed to establish standard CSA of the nerves in the IAC. In contrast to our study, all measurements were obtained manually. Method of image acquisition also varied from 1.5T MRI using 3D FIESTA (13) to 3T MRI CISS or driven equilibrium sequence. Due to this, comparability between studies seems to be limited. However, in all studies CN CSA was bigger compared with FN CSA. So, although a standard size of CSA of the nerves in the IAC seems not to be available, due to the differences in the way measurements are obtained or calculated, and image acquisition is facilitated; this seems to be a robust advice to radiologists, seeking to detect CN hypoplasia or degeneration in comparing CN CSA with FN CSA within their own imaging system. This seems at least to be true in our setting. FN CSA in our study was 1.04 ± 0.03 mm^2^. CN CSA in patients with pantonal hearing loss of more than 70 dB was 0.96 ± 0.06 mm^2^, patients with complete deafness showed even less CN CSA with 0.97 ± 0.07 mm^2^ (Fig. 7A), therefore being considerably smaller compared with average FN CSA. The same could be observed in patients with left corner hearing loss (Fig. 7B), with a CN CSA of 0.98 ± 0.06 for score 5 patients. A similar effect can be observed with Peng et al. (1), where the average CN CSA in patients with adult auditory neuropathy syndrome and patients with SNHL is consistently small, compared with average FN CSA.

**TABLE 3.** Cross-sectional area (mm²) of facial, vestibular, and cochlear nerves of healthy patients as reported here (Reimann et al.) and compared to the literature

Cross-sectional area (mm²)								
	Reimann et al.; mean ± SEM	Peng et al. (1); mean ± SD	Lou et al. (12); mean ± SEM	Polat et al. (13); mean ± SD	Nakamichi et al. (11); mean ± SD	Kang et al. (7); mean ± SD	Herman and Angeli (6); mean ± SEM	Jaryszak et al. (14); mean ± SD
MRI and sequence	3T MRI, 3D, ZOOMIT	3T MRI, 3D, FIESTA	3T MRI, 3D, FIESTA	1.5T MRI, 3D, FIESTA	3T MRI, 3D, CISS	3T MRI, 3D, DRIVE	3T MRI, 3D, CISS	MRI, 3D CISS
Facial nerve	1.04 ± 0.03 (n = 155)	0.50 ± 0.12 (n = 48)	—	0.49 ± 0.24 (n = 158)	0.83 ± 0.27 (n = 172)	0.79 ± 0.31 (n = 144)	—	—
Vestibular nerve	1.78 ± 0.05 (n = 156)	—	—	0.87 ± 0.37 (n = 158)	—	—	—	—
Cochlear nerve	1.23 ± 0.11 (n = 10)	0.70 ± 0.10 (n = 48)	1.12 ± 0.08 (n = 168)	0.64 ± 0.37 (n = 158)	1.07 ± 0.30 (n = 172)	0.98 ± 0.33 (n = 144)	0.94 ± 0.28 (n = 14)	1.10 ± 0.26 (n = 45)

3D indicates 3-dimesional; CISS, constructive interference in steady state; DRIVE, driven equilibrium; FIESTA, fast imaging employing steady-state acquisition; SEM, standard error of the mean; T, tesla; ZOOMIT, zoomed imaging technique.

Our results show that there is no gender-related difference in CSA of FN and CN. This is in line with the result of Kang et al., who showed that in a cohort of Korean patients no gender-related significant difference in FN/CN ratio diameters and CSA exist. Similarly, Nakamichi et al. found no difference regarding age and gender in CSA of CN and FN. These results are corroborated by a cadaver study of Nadol and Xu (5,7,11). We found a significantly smaller CSA of the VN in women compared with male. This has so far not been reported in literature and seems to be lacking a pathophysiological correlation.

Several authors have shown that there is no correlation between CN or FN CSA and age (7,11,12). For the FN, we can confirm this, using our 3D pTX T2 SPACE sequence and semiquantitative CSA evaluation. Due to our small sample size with normal hearing patients, we cannot make this assumption based on our data for the CN. However, at least 3 studies (7,11,12) independently showed this for the CN CSA. More important, we can show for the first time that VN CSA is significantly declining with age. As dizziness and vertigo is more common in the elderly (presbyvertigo), this might be attributed to the reduced VN CSA. However, it remains unclear if nerve degeneration is a result of loss of vestibular function, or if nerve degeneration is a clinical sign on its own. Further studies are needed to address this more clearly.

Kang et al. (7) found a significant difference in horizontal diameter of CN in ears with hearing loss, in comparison with normal hearing ears. However, their results are restricted, as they included many patients with sudden SNHL. In patients with auditory neuropathy syndrome, as well as SNHL, CN CSA was significantly reduced compared with normal hearing patients (1). Our results show that in patients with long-term hearing loss (10 years or longer), CN CSA is decreasing depending on the severity of either pantonal or left corner hearing loss; concluding: the more severe the hearing loss the smaller the CN CSA.

The retrospective character of our study limits its clinical implications, as we cannot derive the benefit of hearing rehabilitation with, for example, cochlear implant in our patient group. In adults with long-term hearing loss, auditory deprivation of the brain seems to be a limiting factor for reaching open speech perception after CI. However, in children with hypoplasia of the CN and CI, an impaired outcome regarding speech understanding has previously been reported (10,15). As indication for CI has been pushing the boundary over the last years, evaluation of CN CSA might contribute to clinical decision making before implantation. However, further studies are needed to precisely determine the influence of CN CSA on open speech perception with CI.

To the best of our knowledge, this is the first study measuring CN, VN, and FN diameter, using a semiautomatic algorithm and a 3T MRI pTX T2 SPACE sequence. We show that the semiautomatic segmentation algorithm can be used to quantify nerve diameter, and discriminate patients with different severity of hearing loss according to their CN CSA. Comparison to FN CSA can be a helpful tool for the radiologist to assess CN degeneration within their individual imaging system. Additionally, we report a decrease in VN CSA in older patients. However, it remains unclear if nerve degeneration is a result of loss of vestibular function or if nerve degeneration is a clinical sign on its own.

## FUNDING SOURCES

This work was supported by 2339-0-0/Fortüne Program, University of Tübingen, Tübingen, Germany to Katrin Reimann.

## CONFLICT OF INTEREST STATEMENT

None declared.

## DATA AVAILABILITY STATEMENT

The datasets generated during and/or analyzed during the current study are not publicly available, but are available from the corresponding author on reasonable request.
